# Self-powered acceleration sensors arrayed by swarm intelligence for table tennis umpiring system

**DOI:** 10.1371/journal.pone.0272632

**Published:** 2022-10-17

**Authors:** Ke Lu, Chaoran Liu, Haiyang Zou, Yishao Wang, Gaofeng Wang, Dujuan Li, Kai Fan, Weihuang Yang, Linxi Dong, Ruizhi Sha, Dongyang Li

**Affiliations:** 1 Ministry of Education Engineering Research Center of Smart Microsensors and Microsystems, College of Electronics and Information, Hangzhou Dianzi University, Hangzhou, China; 2 School of Materials Science and Engineering, Georgia Institute of Technology, Atlanta, GA, United States of America; 3 State Key Laboratory for Manufacturing Systems Engineering, System Engineering Institute, Xi’an Jiaotong University, Xi’an, China; 4 Department of Electronics and Information Engineering, Tongji University, Shanghai, China; UCSI University, MALAYSIA

## Abstract

Table tennis competition is voted as one of the most popular competitive sports. The referee umpires the competition mainly based on visual observation and experience, which may make misjudgments on competition results due to the referee’s subjective uncertainty or imprecision. In this work, a novel intelligent umpiring system based on arrayed self-powered acceleration sensor nodes was presented to enhance the competition accuracy. A sensor node array model was established to detect ball collision point on the table tennis table. This model clearly illuminated the working mechanism of the proposed umpiring system. And an improved particle swarm optimization (level-based competitive swarm optimization) was applied to optimize the arrayed sensor nodes distribution by redefining the representations and update rules of position and velocity. The optimized results showed that the number of sensors decreased from 58 to 51. Also, the reliability of the optimized nodes distribution of the table tennis umpiring system has been verified theoretically. The results revealed that our system achieved a precise detection of the ball collision point with uniform error distances below 3.5 mm. Besides, this research offered an in-depth study on intelligent umpiring system based on arrayed self-powered sensor nodes, which will improve the accuracy of the umpiring of table tennis competition.

## Introduction

As one of the hot events in the Olympic Games, table tennis competition has got increasing attention from all countries. However, the umpire in table tennis competition is still entirely determined by the professional ability and subjective judge of the referee [1, 2]. Table tennis competition has the characteristics of fast speed and small ball. It is hard for the referee to make accurate umpire when an edge ball or net ball appears [3]. Furthermore, in a highly strained mental state, professional referees are also prone to mental fatigue, which may lead them to make misjudgments. The collision point of table tennis on the tabletop is a key reference factor for referee to make the final umpire [4]. Therefore, precise positioning of collision point will effectively improve the accuracy of judgment.

Hawk-eye technology can be introduced into table tennis competition to accurately locate the collision point (CP) on the table caused by a ball, thereby reducing misjudgments. But it will cause long pauses, which may impact athlete’s state [5]. Sensor network has been proved as an effective positioning system with potential applications in many fields, such as military affairs [6–8] and civil engineering [9–11]. To improve the sensing efficiency of the sensor and save costs, the sensors distribution optimization has been presented. Many approaches have been developed to optimize the sensors deployment, in which the sensors distribution based on particle swarm optimization (PSO) gains wide attentions [12–19]. PSO was first proposed by Eberhart and Kennedy [20] in 1995 to solve optimization problems. It is a population-based algorithm inspired from the behaviors of social animals such as birds. The individual in PSO is called particle and each particle has a position and velocity. The position of a particle is viewed as a candidate solution for the target optimization problem. Owing to simplicity and efficiency, PSO has become a vital tool to solve optimization problems [21, 22]. It has been successfully implemented to solve various optimization problems for decades, especially for those intractable NP-hard issues [23]. Unfortunately, the classical PSO easily leads to stagnation or premature convergence [24].

In this work, we presented a real-time and accurate intelligent umpiring system based on arrayed self-powered acceleration sensors to assist referee make precise umpiring. Firstly, a model was established to expound the designed umpiring system which was used to detect the collision point of the ball on the table tennis table. To simplify the arrayed sensor nodes, save costs and optimize nodes distribution, we presented a level-based competitive swarm optimization (LCSO). The representations and update rules of position and velocity could be redefined in LCSO. By LCSO algorithm, the number of sensors reduced from 58 to 51. Finally, we verified the reliability of optimized nodes distribution of the table tennis umpiring system theoretically. This research provided a new strategy for umpire of table tennis competitions, which would make umpiring precise and real-time.

## Model

When two dissimilar materials come into contact, their surfaces will generate positive and negative electrostatic charges due to the contact electrification respectively. When the two materials are separated due to the action of mechanical force, the positive and negative charges generated by the contact electrification are also separated. And this charge separation will correspondingly generate an induced potential difference between the upper and lower electrodes of the material. If a load is connected between the two electrodes or the two electrodes are short circuit, the induced potential difference will drive electrons to flow between the two electrodes through an external circuit—this is triboelectric nanogenerator (TENG).

**Fig 1:**
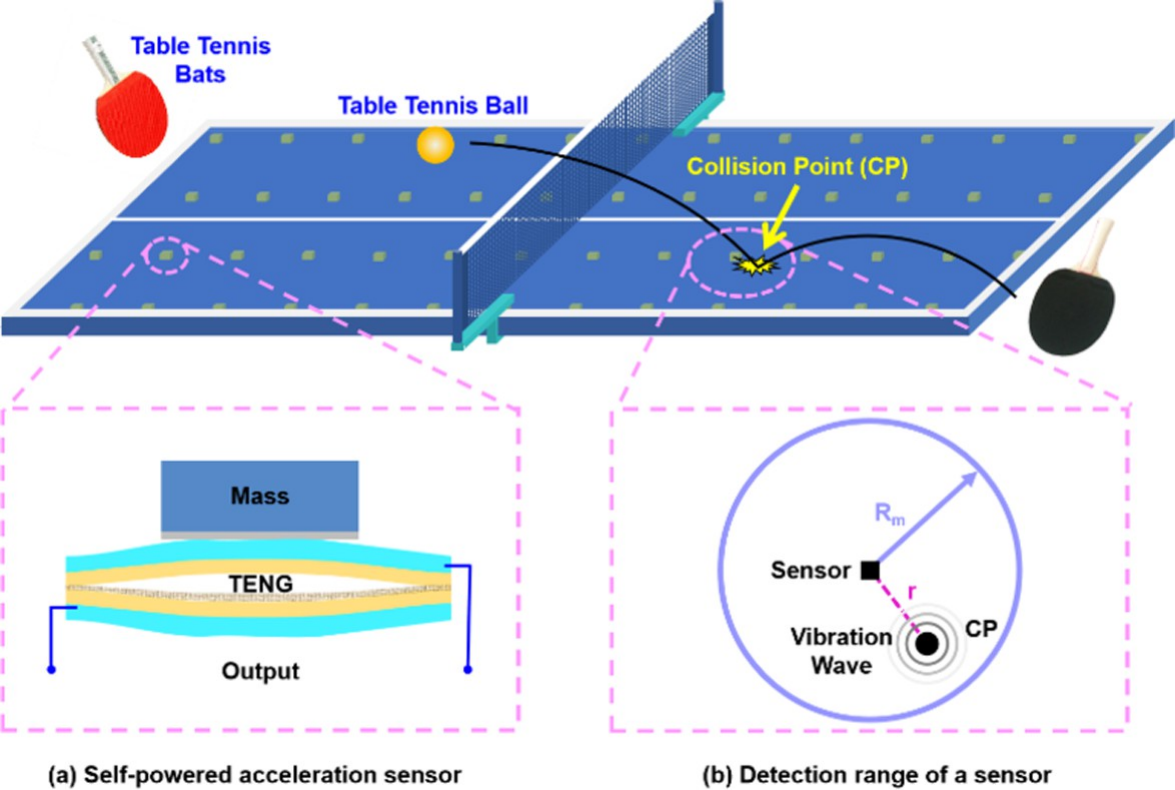
Schematic of the intelligent umpiring system by deploying the self-powered acceleration sensor on the back of the table. (a) Schematic of the self-powered acceleration sensor. (b) Detection range of a sensor with a maximum detection distance.

The referee umpires the score mainly based on the ball collision point (CP) on the tabletop [5] in a table tennis competition. It is hard for human eyes to precisely umpire when touching net or edge ball appears. Here, we present an intelligent collision point positioning system by distributing the acceleration sensor on the back surface of the table (Fig 1). We prefer the self-powered acceleration sensor [25–27] based on TENG to overcome battery shortcomings such as limited power storage, maintenance required, and large weight. The bottom-up components of the sensor consist of a TENG, an insulating layer, and a mass (Fig 1A). As the table tennis hits the tabletop, the acceleration sensor will detect a tabletop vibration wave and output a voltage signal.

**Fig 2:**
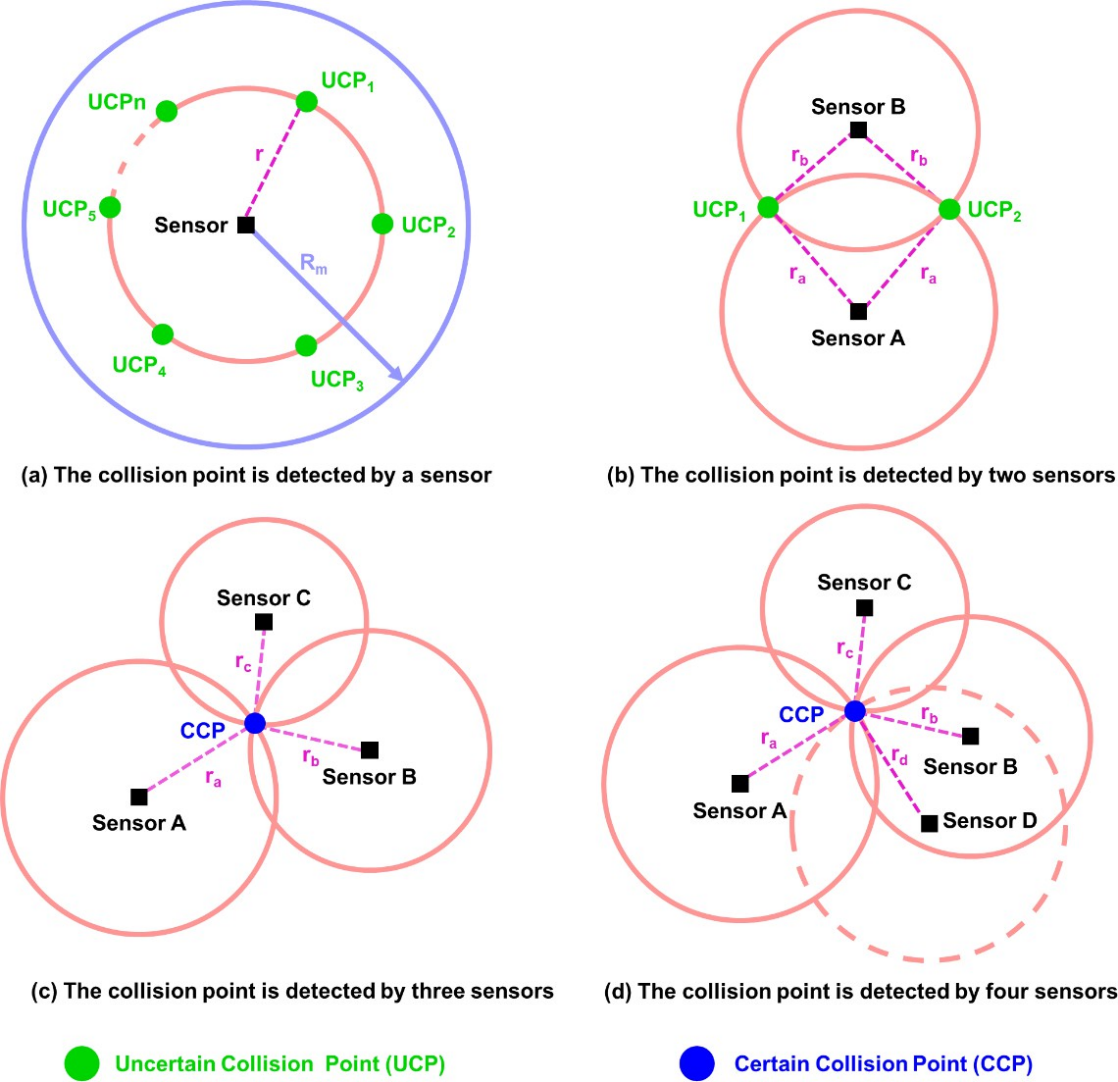
The possible location of the collision point when the point is detected by (a) a sensor, (b) two sensors, (c) three sensors and (d) four sensors at the same time, respectively.

The sensor has a maximal detection distance (*R*_m_). Within the detection range of the sensor, the vibration of any collision point can be detected (Fig 1B). Obviously, more than three sensor nodes are required to uniquely determine a collision point on a two-dimensional plane. One sensor cannot determine the location of the collision point (Fig 2A), while two sensors have a 50% chance to accurately locate the collision point (Fig 2B). Four or more sensors are wasteful, complicating the system and increasing cost (Fig 2D). Therefore, three sensors are the best.

The sensor nodes distribution should be optimized to simplify and efficiently detect all possible collision points (CPs) on the entire tabletop. Assuming that infinitely many sensor nodes are installed on the back of the table, a CP position must be located inside a triangle formed by the three nearest sensors. And the distances between the CP and each of the three nearest sensors are less than *R*_m_ of sensor. We define the three nearest sensors as the detection sensor set (DSS). It is obvious that different CPs may have the same DSS. And these CPs with the same DSS will completely fill in the triangle formed by their DSS. Based on the assumption, we regard the tabletop as a 2-D rectangle composed of triangles. When we try to expand these triangles synchronously, the number of sensors installed on the back of the table will gradually decrease. The triangle cannot be expanded infinitely because the distance between any CP in triangle and vertexes must be less than *R*_m_. Therefore, the sensor distribution optimization can be converted as the optimization of the triangle area.

**Fig 3:**
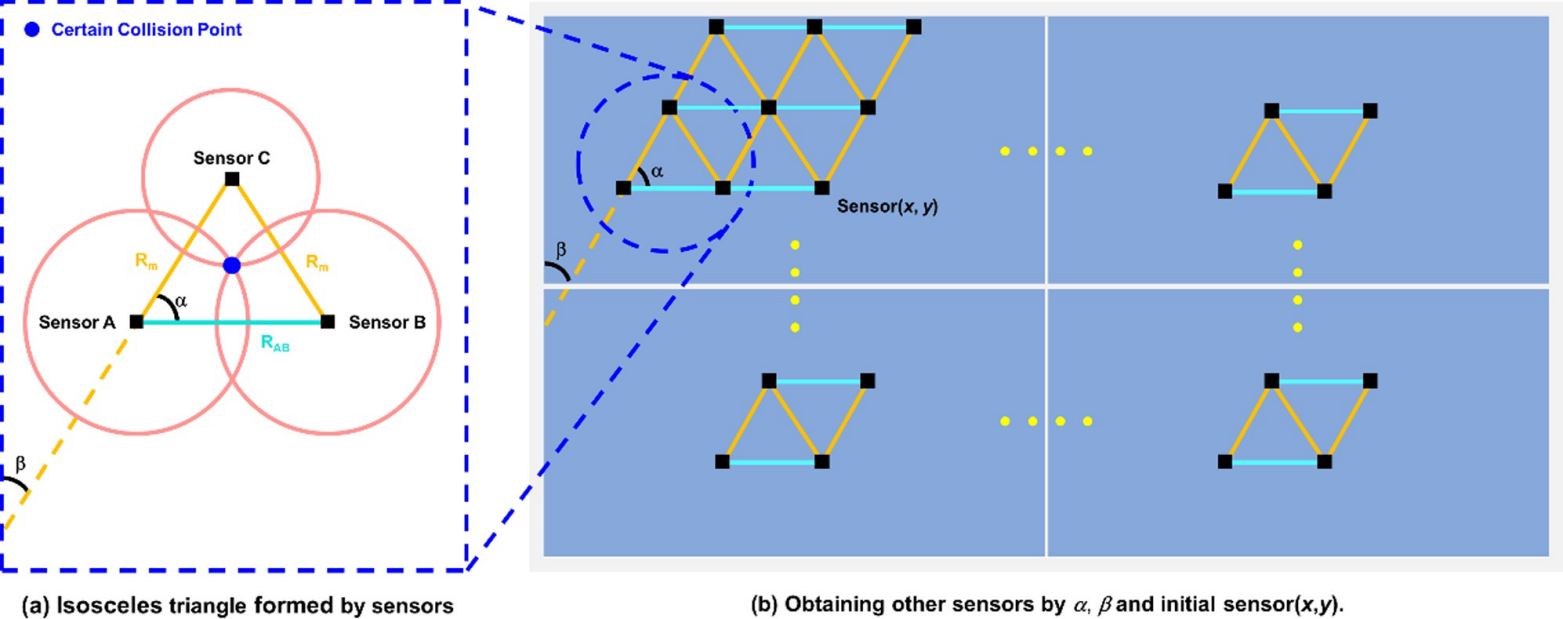
(a) Isosceles triangle formed by the three nearest sensors of the collision point. (b) Obtaining the position of all sensors in the table by *α*, *β* and initial sensor(*x*,*y*).

Commonly, the distances between any point in a triangle and vertexes are less than the length of the triangle longest edge. The triangles cannot cover the maximum area on the tabletop if they are not isosceles triangles. Therefore, we reduce the number of sensor nodes by optimizing the shape of isosceles triangle. Furthermore, the included angles *α* and *β* were explored to optimize the sensor distribution (Fig 3A).

Based on the above theories, the sensor distribution optimization can be modeled as a function: *minsensor* = *f* (*α*, *β*, *x*, *y*). *α* is the base angle of the isosceles triangle, which determines the area of the triangle. *β* is the included angle between the waist of the isosceles triangle and the edge of the table (Fig 3A). *x* and *y* are abscissa and ordinate of the initial sensor respectively. With the function *minsensor*, we will get the positions of other sensors and the number of sensors inside the table after obtaining the angles of *α* and *β* and the abscissa *x* and ordinate *y* of initial sensor (Fig 3B). The pseudo code of the function *minsensor* = *f* (*α*, *β*, *x*, *y*) is shown as following:

## Algorithm 1: The function *minsensor* = *f* (*α*, *β*, *x*, *y*)

**Coordinate system:** Define the lower left of the table tennis table as the origin and the edge of the table as the coordinate axis to establish a rectangular coordinate system. (0 mm ≤ *x* ≤ 2740 mm and 0 mm ≤ *y* ≤ 1525 mm)
**Input:** base angle *α*, intersection angle *β*, the initial sensor(*x*,*y*)
**Output:** the number of sensors inside the table tennis table
 //*sensors*: store the coordinates of all sensors located inside the table;
01: Define global variables: *sensors*;
 //*U* denotes a set of *sensors*;
02: *U* = *Ø;*
03: Store initial sensor(*x*,*y*) into *U*;
04: **while** *U* ≠ *Ø* **do**
05: randomly select a sensor(*x*,*y*) from *U*;
06: remove sensor(*x*, *y*) from *U*;
  //The sensor cannot be located outside the table
07: **if** x > 2740 **or** x < 0 **or** y > 1525 **or** y < 0 **then**
08:  **if** *x* > 2740 **then**
09:   *x* = 2740;
10:  **end if**
11:  **if** *x* < 0 **then**
12:   *x* = 0;
13:  **end if**
14:  **if** *y* > 1525 **then**
15:   *y* = 1525;
16:  **end if**
17:  **if** *y* < 0 **then**
18:   *y* = 0;
19:  **end if**
20:  **if** sensor(*x*, *y*) is not the member of *sensors* **then**
21:   Store sensor(*x*, *y*) into *sensors*;
22:  **end if**
23: **else**
24:  **if** sensor(*x*, *y*) is not the member of *sensors* **then**
25:   Store sensor(*x*, *y*) into *sensors*;
26:   Calculate the coordinates of the six sensors around sensor(*x*, *y*) based on *α*,*β* and *R*_*m*_;
27:   Store these six sensors into *U*;
28:  **end if**
29: **end if**
30: **end while**

31: **if** sensor(0, 0) is not the member of *sensors* **then**

32: Store sensor(0, 0) into *sensors*;
33: **end if**
34: **if** sensor(0, 1525) is not the member of *sensors* **then**
35: Store sensor(0, 1525) into *sensors*;
36: **end if**
37: **if** sensor(2740, 0) is not the member of *sensors* **then**
38: Store sensor(2740, 0) into *sensors*;
39: **end if**
40: **if** sensor(2740, 1525) is not the member of *sensors* **then**
41: Store sensor(2740, 1525) into *sensors*;
42: **end if**
43: **return** *sensors and* the size of *sensors*;

## Level-based competitive swarm optimizer

The idea of particle swarm optimization (PSO) originates from the movement of birds, where PSO can search the decision space for promising solutions just like birds searching for food [28]. Each particle in PSO has two attributes (position and velocity). These two attributes represent current feasible solution and moving direction of the particles, respectively. In the optimization process, the velocity and position of each particle are iteratively updated by using the following equations:

$$\begin{array}{l} V_{i}\left(t+1\right)=\omega V_{i}\left(t\right)+c_{1}r_{1}\left(t\right)\left(Pbest_{i}\left(t\right)-X_{i}\left(t\right)\right)\\ +c_{2}r_{2}\left(t\right)\left(Gbest\left(t\right)-X_{i}\left(t\right)\right) \end{array}$$ (1)

$$X_{i}\left(t+1\right)=X_{i}\left(t\right)+V_{i}\left(t+1\right)$$ (2)

Where *t* is the current number of iterations, $X_{i}=(x_{i}^{1},x_{i}^{2},\ldots ,x_{i}^{D})$ and $V_{\text{i}}=(v_{i}^{1},v_{i}^{2},\ldots ,v_{i}^{D})$ are the position and the velocity of the *i-th* particle, respectively. $Pbest_{i}=(pbest_{i}^{1},pbest_{i}^{2},\ldots ,pbest_{i}^{D})$ is the best position where *i-th* has achieved so far, *Gbest* = (*gbest*^1^, *gbest*^2^,…,*gbest*^*D*^) represents the current best position in the particles swarm. *D* denotes the dimension size of the objective function, *ω* is termed as the inertia weight [29]. *c*_1_ and *c*_2_ are acceleration coefficients adjusted by users. *r*_1_ and *r*_2_ are two random number generated within (0, 1).

Refer to Eqs (1) and (2), a particle iteratively updates its position and traverses the search space to seek the potential optimum of the objective function. However, this learning strategy commonly causes stagnation and premature convergence. The reason is that PSO shows weak capacity of diversity preservation [30]. To address this issue, a large amount of improvements on PSO can be found in literatures [31–39]. The current methods can be mainly classified into the following two categories, adopting cooperatively coevolutionary framework and designing new learning strategies. Methods in the first category focus on the large-scale optimization problems, which divides the original problem into several simultaneous and independently optimized sub-problems, such as the random variable grouping-based algorithms [40], differential grouping-based algorithms [41, 42] and machine learning based algorithms [43]. However, such methods commonly have the problems with high computational cost and inaccurate variable grouping. Methods in the second category aim to improve the search ability of PSO under different learning strategies, including the comprehensive learning and competitive learning for diversity enhancement [31], adaptive parameter adjustments and level-based learning [44] for balancing the convergence and diversity [45]. Nevertheless, these methods still cannot help PSO to effectively address complex problems, since the diversity preservation continue challenging PSO.

Among these methods, CSO [46] and DLLSO [44] are two competitive algorithms proposed in recent years to address optimization problems with large scale dimensionality. With a deep insight into these two algorithms, we find that CSO and DLLSO show several special advantages when comparing with PSO. CSO selects the exploitation exemplars by using a pairwise competition mechanism. And it only updates half of the particles swarm at each generation, which can maximize the diversity of the exemplars and preserve the promising solutions. On the other hand, the level-learning strategy proposed in DLLSO can further diverse the exploration exemplars when compared with CSO. Inspired by CSO and DLLSO, a novel particle swarm optimization method is proposed in this paper. In the proposed method, neither the personal best position of each particle (*pbest*) nor the global best position (*gbest*) is involved in updating the particles swarm. Conversely, we introduce a level-based pairwise competition mechanism. In general, the particles swarm will be divided into several levels according to the fitness, similar to the design in DLLSO. Therefore, the particles in higher levels hold more beneficial information which is contributing to guide the particles swarm toward the global optimum area [47, 48]. More specifically, the proposed pairwise competition mechanism is executed in each level to distinguish the particles that need to be updated and the corresponding exploration exemplars. On the other hand, the particles in higher levels should be selected as the exploitation exemplars to guide particles in lower levels to search the solution space for exploitation.

**Fig 4:**
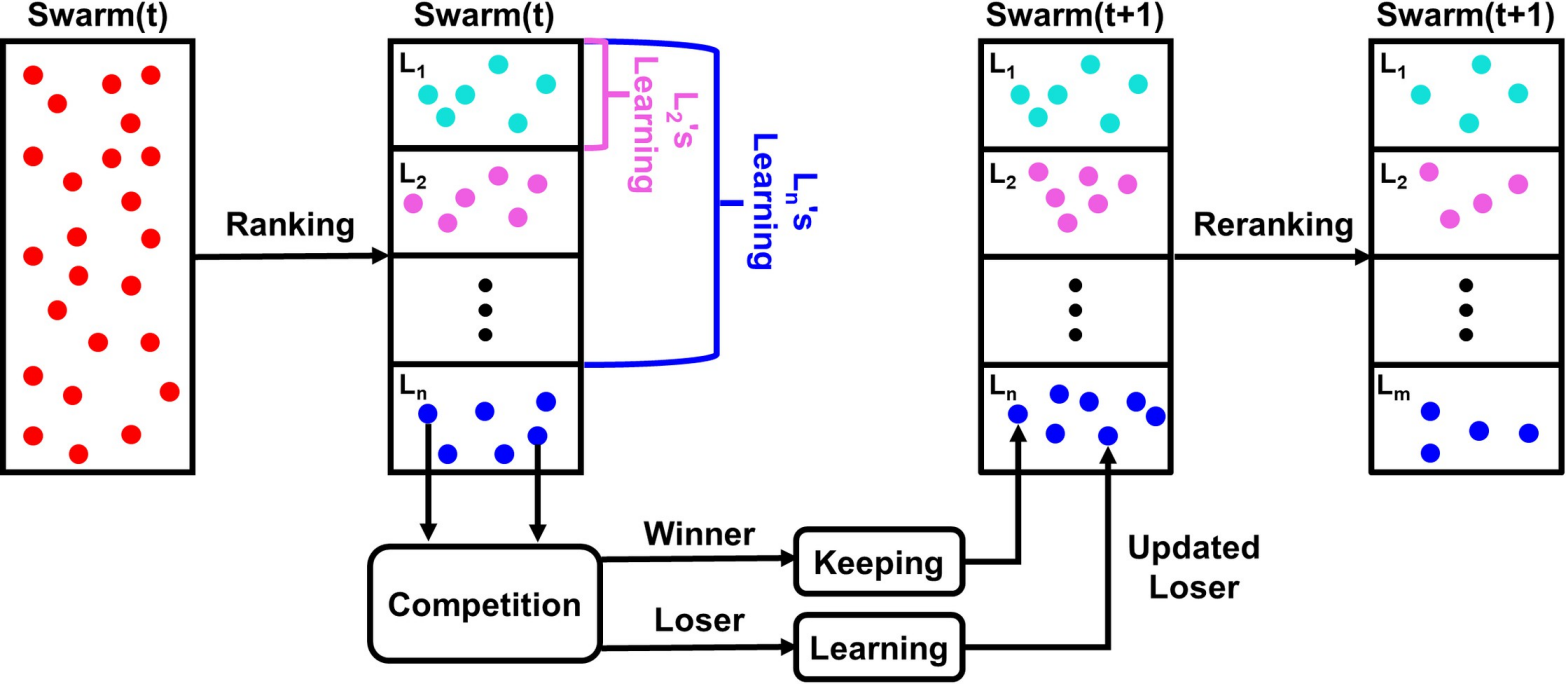
Framework of the proposed level-based competitive swarm optimization.

The framework of LCSO is shown in Fig 4. Firstly, particles in the swarm are sorted by fitness in ascending order and divide into several levels. The better particles belong to higher levels and all levels have the same number of particles (*L*_1_ is the highest level). Lower-level particles can learn from higher-level particles.

The number of candidate exemplars for particles in higher level is fewer, which matches the expectation that better particles should do more exploitation rather than exploration. Then, in each level, two particles are randomly chosen to contest until all particles have participated in at least one competition. After each competition, the winner that having a better fitness will be passed directly to the next generation particles swarm, and the loser will update its position and velocity by learning from the winner and higher-level particles. Since the particles in the first level are the best of the whole particles swarm in the current generation, better solutions are usually found near these ones. The particles in the highest level directly enter the next generation. The position and velocity of the particle that loses the competition will update as follows:

$$\begin{array}{l} V_{i,j}\left(t+1\right)=r_{1}(t)V_{i,j}\left(t\right)+r_{2}\left(t\right)\left(X_{m,n}\left(t\right)-X_{i,j}\left(t\right)\right)\\ +\phi r_{3}\left(t\right)\left(X_{i,k}\left(t\right)-X_{i,j}\left(t\right)\right) \end{array}$$ (3)

$$X_{i,j}\left(t+1\right)=X_{i,j}\left(t\right)+V_{i,j}\left(t+1\right)$$ (4)

Where *t* is the iteration number, $X_{i,j}=(x_{i,j}^{1},x_{i,j}^{2},\ldots ,x_{i,j}^{D})$ and $V_{i,j}=(v_{i,j}^{1},v_{i,j}^{2},\ldots ,v_{i,j}^{D})$ are the position vector and the velocity vector of the *j-th* loser particle from the *i-th* level (*L*_*i*_) respectively. $X_{m,n}=(x_{m,n}^{1},x_{m,n}^{2},\ldots ,x_{m,n}^{D})$ is the randomly selected exemplars from *m-th* level (*L*_*m*_) (*m*∈[1,*i*-1]), and $X_{i,k}=(x_{i,k}^{1},x_{i,k}^{2},\ldots ,x_{i,k}^{D})$ represents the winner after competing with *X*_*i*,*j*_. *r*_1_(*t*), *r*_2_(*t*), and *r*_3_(*t*) are three random variables in the range [0, 1] and *ϕ* is the control parameter within [0, 1] which is in charge of the influence of the second exemplar.

The pseudo code of LCSO is outlined in Algorithm 2.

## Algorithm 2: Framework of LCSO

**Input:** swarm *P*, swarm size *NP*, number of levels *N*, level size *LS*, maximum number of fitness evaluations *MAX_FES*.
**Output:** the final solution *x* and its fitness *f(x)*
//*t* denotes the generation number;
01: *t* = 0;
02: *fes* = 0;
03: Initialize the swarm *P*(0) randomly;
04: **while** *fes* < *MAX_FES* **do**
05: calculate the fitness of all particles in *P*(*t*);
06: *fes* + = *NP*;
//At each generation, *N* may different;
07: Sort particles in ascending order of fitness and divide them into *N* levels;
//Update particles in *L*_*N*_,…, *L*_*2*_;
08: **for** i = {*N*,…, 2} **do**
**//***U*_*i*_ denotes a set of *i-th* level particles that have not yet participated in a competition;
09: **while** *U*_*i*_ ≠*Ф* **do**
10: randomly choose two particles *X*_*1*_*(t)*, *X*_*2*_*(t)* from the *i-th* level *L*_*i*_;
11: **if** *f(X*_*1*_*(t))* < *f(X*_*2*_*(t))* **then**
12: *X*_*w*_*(t)* = *X*_*1*_*(t)*, *X*_*l*_*(t)* = *X*_*2*_*(t)*;
13: **else**
14: *X*_*w*_*(t)* = *X*_*2*_*(t)*, *X*_*l*_*(t)* = *X*_*1*_*(t)*;
15: **end if**
16: add *X*_*w*_*(t)* into *P* (*t* + 1);
17: Select a level from the top (*i*-1) levels: *L*_*m*_;
18: Randomly select a particles from *L*_*m*_: *X*_*m*,*n*_;
19: Update particle *X*_*l*_*(t)* refer to (3) and (4);
20: add the updated *X*_*l*_(*t*) to *P* (t + 1);
21: remove *X*_*1*_*(t)*, *X*_*2*_*(t)* from *U*_*i*_;
22: **end while**
23: **end for**
24: add the first-level particles into *P* (*t* + 1);
25: *t* = *t* + 1;
26: **end while**

The difference between LCSO and PSO and its other variants is the proposed level-based pairwise competition mechanism (LCM) of LCSO. Firstly, LCM divides the swarm into several levels based on particles’ fitness. Then, for each particle that lose the pairwise competition, two exemplars are selected from higher level particles and the winner of the pairwise competition to guide its updating for exploitation and exploration.

The complexity, exploration and exploitation of LCSO are theoretically analyzed as follows.

### Computational complexity

Since the algorithm usually takes the number of fitness evaluations as the final criterion, this paper assumes using the same swarm size N to compare the computational complexity per generation between the proposed algorithm and PSO. Algorithm structure, computation cost at each generation and the required memory cost are taken into consideration to compare the computational complexity of LCSO and PSO.

Firstly, LSCO has the same structure as PSO, which is easy to implement. Secondly, the extra computational cost of LCSO at each generation are generated in Lines 7–18 in Algorithm 2 to divide the swarm into N levels and execute competition, which both cost O(N). In summary, LCSO only introduces O(2N) extra computational cost at each generation compared with PSO. Last but not least, LCSO has greater advantage in memory cost when comparing with PSO as it does not need to store the historical best particles.

In summary, LCSO is superior in terms of implementation and computational complexity.

### Exploration ability

Exploration is crucial to Evolutionary Algorithms (EAs) since it is conducive to find more promising areas in the decision space to avoid premature convergence. One way to enhance the exploration ability of EAs is maintenance of swarm diversity, which can be achieved by diversing the exemplars of particles. The exploration exemplars in LCSO are selected by using a pairwise competition, indicating that all updated particles have different exploration exemplars. However, the exploration exemplars in DLLSO are randomly selected. Therefore, it can be concluded that the exploration exemplars in LCSO have more diversity than that in DLLSO, CSO and the canonical PSO.

Further, we rewrite Eq (3) as Eqs (5–7):

$$V_{i,j}\left(t+1\right)=r_{1}(t)V_{i,j}\left(t\right)+\theta_{1}\left(p_{1}-X_{i,j}\left(t\right)\right)$$ (5)

$$\theta_{\text{1}}\text{=}r_{2}(t)+\phi r_{3}(t)$$ (6)

$$p_{1}=\frac{r_{2}(t)}{r_{2}(t)+\phi r_{3}(t)}X_{m,n}\left(t\right)+\frac{\phi r_{3}(t)}{r_{2}(t)+\phi r_{3}(t)}X_{i,k}\left(t\right)$$ (7)

Similarly, the equation of updated velocity of classical PSO, CSO and DLLSO can be rewritten to (8), (9), (10) respectively.


$$\begin{array}{c} V_{i}\left(t+1\right)=\omega V_{i}\left(t\right)+\theta_{2}\left(p_{2}-X_{i}\left(t\right)\right)\\ \theta_{2}=c_{1}r_{1}(t)+c_{2}r_{2}(t)\\ p_{\text{2}}=\frac{c_{1}r_{1}(t)}{c_{1}r_{1}(t)+c_{2}r_{2}(t)}Pbest_{i}\left(t\right)+\frac{c_{2}r_{2}(t)}{c_{1}r_{1}(t)+c_{2}r_{2}(t)}Gbest(t) \end{array}$$ (8)

$$\begin{array}{c} V_{l}\left(t+1\right)=r_{1}(t)V_{l}\left(t\right)+\theta_{3}\left(p_{3}-X_{l}\left(t\right)\right)\\ \theta_{\text{3}}\text{=}r_{2}(t)+\phi r_{3}(t)\\ p_{\text{3}}=\frac{r_{2}(t)}{r_{2}(t)+\phi r_{3}(t)}X_{w}\left(t\right)+\frac{\phi r_{3}(t)}{r_{2}(t)+\phi r_{3}(t)}\bar{X}\left(t\right) \end{array}$$ (9)

$$\begin{array}{c} V_{i,j}\left(t+1\right)=r_{1}(t)V_{i,j}\left(t\right)+\theta_{\text{4}}\left(p_{\text{4}}-X_{i,j}\left(t\right)\right)\\ \theta_{\text{4}}\text{=}r_{2}(t)+\phi r_{3}(t)\\ p_{\text{4}}=\frac{r_{2}(t)}{r_{2}(t)+\phi r_{3}(t)}X_{rl_{1},k_{1}}\left(t\right)+\frac{\phi r_{3}(t)}{r_{2}(t)+\phi r_{3}(t)}X_{rl_{2},k_{2}}\left(t\right) \end{array}$$ (10)

Where $X_{w}=(x_{w}^{1},x_{w}^{2},\ldots ,x_{w}^{D})$ is the position of the winner and $\bar{X}=({\bar{x}}^{1},{\bar{x}}^{2},\ldots ,{\bar{x}}^{D})$ is the average position of the particles swarm in CSO. And in DLLSO, $X_{rl1,k1}=(x_{rl1,k1}^{1},x_{rl1,k1}^{2},\ldots ,x_{rl1,k1}^{D})$ randomly selected from *rl1-th* level (*L*_*rl*1_) and $X_{rl2,k2}=(x_{rl2,k2}^{1},x_{rl2,k2}^{2},\ldots ,x_{rl2,k2}^{D})$ randomly selected from *rl2-th* level (*L*_*rl*2_) are the two selected exemplars.

From Eqs (5), (8), (9) and (10), the difference between *p*_*i*_ (*i* = 1, 2, 3, 4) and the particle to be updated is the main source of diversity. As for classical PSO (*p*_2_), *Pbest* of each particle is updated only when the particle finds a better position and *Gbest* is updated only when the swarm finds a better position. It indicates that *Pbest* and *Gbest* may be constant for many generations, which limits exploration ability. In CSO (*p*_3_), the mean position of the swarm is employed as the exploration exemplar of all the particles. Although the mean position is updated at each generation, it is shared by all particles, which is not beneficial for further diversity enhancement. For DLLSO (*p*_4_) based the level-learning strategy, the exploration exemplar *X*_*rl*2,*k*2_ is randomly selected from higher levels for each particle in lower levels. However, a higher-level particle may be chosen as the exploration exemplar of multiple lower-level particles. In our proposed LCSO (*p*_1_), a level-based pairwise competition mechanism is utilized to select the exploration exemplars, where the exploration exemplars are the winners of each pairwise competition in the same level. This mechanism can ensure that all the exploration exemplars are different.

In summary, the diversity preservation ability of the proposed LCSO is better than DLLSO, CSO, and PSO, which benefits for strengthening the exploration ability.

### Exploitation ability

Exploitation is a key point for swarm to refine promising areas, especially when computational resources are limited. Generally, the exploitation ability can be reflected by the difference between the exploitation exemplars and the corresponding updated particles.

The first exemplars in LCSO and DLLSO are the exploitation exemplars, which are randomly selected from the higher level. The differences of the exploitation exemplar selections between LCSO and DLLSO are presented as follows. The exploitation exemplar for a particle in DLLSO is selected from a level that at least two levels higher than the updated particle. However, the exploitation exemplars in LCSO are selected from any higher level. This indicates that LCSO can exploit the exploitation exemplars within a smaller difference when comparing with DLLSO, which is potential in the exploitation of a local area. Similar to the analyses in [39], LCSO theoretically has a faster convergence ability than CSO and classical PSO.

The above analyses clearly verify that LCSO shows superior exploration and exploitation abilities.

To verify the superiority and efficiency of the proposed LCSO, we have compared LCSO with five representative PSO variants [37, 44–46, 49] by a series of experiments conducted on the CEC’2013 benchmark sets [50] (Table 1). For fairness, the key parameters in each algorithm are set according to the suggestions in the corresponding papers. The symbols above the p values, “+”, “-”, and “=“, mean that the performance of LCSO is significantly better than, worse than, or statistically equivalent to the compared algorithm on the corresponding benchmark respectively (Table 2). In addition, the bold p means that LCSO is significantly better than other algorithms. Furthermore, w/l/t at the bottom of the table represents the times that LCSO wins/loses/ties in the competitions compared with the corresponding algorithms. Comparing with five state-of-the arts algorithms, LCSO outperforms the compared algorithms on most of the 28 functions, which clearly demonstrates that the proposed LCSO has a better performance.

**Table 1:** Summary of the 28 CEC’13 test functions.

No.	Functions	Unimodal/Multimodal	Non-separable/Separable
1	Sphere Function	Unimodal	Separable
2	Rotated High Conditioned Elliptic Function	Unimodal	Non-separable
3	Rotated Bent Cigar Function	Unimodal	Non-separable
4	Rotated Discus Function	Unimodal	Non-separable
5	Different Powers Function	Unimodal	Separable
6	Rotated Rosenbrock’s Function	Multimodal	Non-separable
7	Rotated Schaffers F7 Function	Multimodal	Non-separable
8	Rotated Ackley’s Function	Multimodal	Non-separable
9	Rotated Weierstrass Function	Multimodal	Non-separable
10	Rotated Griewank’s Function	Multimodal	Non-separable
11	Rastrigin’s Function	Multimodal	Separable
12	Rotated Rastrigin’s Function	Multimodal	Non-separable
13	Non-Continuous Rotated Rastrigin’s Function	Multimodal	Non-separable
14	Schwefel’s Function	Multimodal	Non-separable
15	Rotated Schwefel’s Function	Multimodal	Non-separable
16	Rotated Katsuura Function	Multimodal	Non-separable
17	Lunacek Bi_Rastrigin Function	Multimodal	--
18	Rotated Lunacek Bi_Rastrigin Function	Multimodal	Non-separable
19	Expanded Griewank’s plus Rosenbrock’s Function	Multimodal	Non-separable
20	Expanded Scaffer’s F6 Function	Multimodal	Non-separable
21	Composition Function 1 (n = 5,Rotated)	Multimodal	Non-separable
22	Composition Function 2 (n = 3,Unrotated)	Multimodal	Separable
23	Composition Function 3 (n = 3,Rotated)	Multimodal	Non-separable
24	Composition Function 4 (n = 3,Rotated)	Multimodal	Non-separable
25	Composition Function 5 (n = 3,Rotated)	Multimodal	Non-separable
26	Composition Function 6 (n = 5,Rotated)	Multimodal	Non-separable
27	Composition Function 7 (n = 5,Rotated)	Multimodal	Non-separable
28	Composition Function 8 (n = 5,Rotated)	Multimodal	Non-separable

**Table 2:** Comparison results of the compared algorithms on CEC’2013 functions with 6 × 105 fitness evaluations.

Function	Quality	CSO	DLLSO	LIPS	HPSO_TVAC	ALC-PSO	LCSO
F1	Mean	2.28E-13	6.37E-13	8.71E-01	2.72E-08	7.47E-12	2.27E-13
	Std	3.74E-16	4.14E-13	2.07E+00	9.92E-09	5.78E-12	1.02E-13
	p-value	**5.00E-03** ^**+**^	**1.38E-08** ^**+**^	**5.35E-10** ^**+**^	**5.35E-10** ^**+**^	**5.25E-10** ^**+**^	-
F2	Mean	3.17E+06	1.76E+07	4.09E+08	2.65E+07	1.32E+08	3.10E+06
	Std	7.43E+05	4.61E+06	1.62E+08	6.23E+06	5.30E+07	8.66E+05
	p-value	5.61E-01 ^=^	**1.42E-09** ^**+**^	**1.42E-09** ^**+**^	**1.42E-09** ^**+**^	**1.42E-09** ^**+**^	-
F3	Mean	1.33E+08	9.15E+09	3.24E+13	2.16E+10	4.64E+10	4.25E+08
	Std	1.40E+08	4.25E+09	3.22E+13	6.23E+09	2.49E+10	3.09E+08
	p-value	6.75E-06^-^	**1.42E-09** ^**+**^	**1.41E-09** ^**+**^	**1.41E-09** ^**+**^	**1.41E-09** ^**+**^	-
F4	Mean	8.76E+04	7.35E+04	2.52E+05	6.66E+04	5.20E+04	2.33E+04
	Std	9.42E+03	1.92E+04	2.14E+04	1.12E+04	8.44E+03	4.54E+03
	p-value	**1.41E-09** ^**+**^	**1.60E-09** ^**+**^	**1.41E-09** ^**+**^	**1.41E-09** ^**+**^	**2.02E-09** ^**+**^	-
F5	Mean	1.14E-13	1.03E-05	14.46E+02	2.77E-04	3.02E-12	3.55E-13
	Std	3.13E-16	6.62E-13	1.78E+01	4.93E-05	4.76E-13	2.70E-13
	p-value	4.16E-11^-^	**7.25E-10** ^**+**^	**7.40E-10** ^**+**^	**7.40E-10** ^**+**^	**7.18E-10** ^**+**^	-
F6	Mean	2.00E+02	2.73E+02	2.95E+03	2.40E+02	6.83E+02	1.99E+02
	Std	3.38E+01	4.16E+01	1.01E+03	4.91E+01	1.55E+02	3.17E+01
	p-value	**1.41E-09** ^**+**^	**1.42E-09** ^**+**^	**1.42E-09** ^**+**^	**1.42E-09** ^**+**^	**1.42E-09** ^**+**^	-
F7	Mean	1.10E+01	6.90E+01	2.66E+03	5.17E+04	1.80E+02	2.00E+00
	Std	2.76E+00	1.48E+01	1.83E+03	4.18E+04	7.01E+01	7.63E+00
	p-value	**1.41E-09** ^**+**^	**1.60E-09** ^**+**^	**1.42E-09** ^**+**^	**1.42E-09** ^**+**^	**1.42E-09** ^**+**^	-
F8	Mean	2.20E+01	2.10E+01	2.20E+01	2.20E+01	2.20E+01	2.10E+01
	Std	3.36E-02	6.31E-02	2.77E-02	2.37E-02	3.20E-02	3.63E-02
	p-value	**5.01E-05** ^**+**^	5.35E-01 ^=^	**1.17E-02** ^**+**^	**1.30E-02** ^**+**^	8.77E-01 ^=^	-
F9	Mean	4.20E+01	5.90E+01	1.18E+02	1.37E+02	1.26E+02	2.70E+01
	Std	4.57E+00	4.23E+00	5.54E+00	5.55E+00	8.02E+00	6.14E+00
	p-value	**1.60E-03** ^**+**^	**2.21E-07** ^**+**^	**1.42E-09** ^**+**^	**1.42E-09** ^**+**^	**1.42E-09** ^**+**^	-
F10	Mean	1.64E-01	2.50E+01	1.37E+03	2.60E+01	1.20E+01	1.28E-01
	Std	1.09E-01	1.12E+01	1.32E+03	6.57E+00	9.21E+00	6.15E-02
	p-value	4.15E-01 ^=^	**1.42E-09** ^**+**^	**1.42E-09** ^**+**^	**1.42E-09** ^**+**^	**1.42E-09** ^**+**^	-
F11	Mean	5.20E+01	1.18E+02	7.45E+02	3.22E+02	2.57E+02	7.20E+01
	Std	7.63E+00	2.23E+01	9.39E+01	5.53E+01	2.55E+01	1.47E+01
	p-value	2.30E-06^-^	**8.28E-09** ^**+**^	**1.41E-09** ^**+**^	**1.41E-09** ^**+**^	**1.41E-09** ^**+**^	-
F12	Mean	8.04E+02	1.40E+02	7.99E+02	1.72E+03	6.70E+02	7.91E+02
	Std	1.87E+01	2.42E+01	1.02E+02	1.57E+02	1.45E+02	1.71E+01
	p-value	**9.90E-03** ^**+**^	1.41E-09^-^	7.415E-01 ^=^	**1.41E-09** ^**+**^	3.31E-04^-^	-
F13	Mean	8.01E+02	3.75E+02	1.30E+03	2.11E+03	1.05E+03	7.84E+02
	Std	1.42E+01	6.19E+01	1.15E+02	1.88E+02	1.58E+02	2.34E+01
	p-value	**3.00E-03** ^**+**^	1.42E-09^-^	**1.42E-09** ^**+**^	**1.42E-09** ^**+**^	**7.38E-09** ^**+**^	-
F14	Mean	1.45E+03	5.19E+03	1.11E+04	3.77E+03	6.58E+03	2.31E+03
	Std	3.34E+02	8.15E+02	5.92E+02	7.13E+02	7.03E+02	6.37E+02
	p-value	4.67E-06-	**2.03E-09** ^**+**^	**1.42E-09** ^**+**^	**1.31E-07** ^**+**^	**1.42E-09** ^**+**^	-
F15	Mean	2.92E+04	9.30E+03	1.41E+04	2.11E+04	2.39E+04	2.88E+04
	Std	5.18E+02	1.50E+03	1.10E+03	4.35E+03	6.12E+03	6.35E+02
	p-value	**4.78E-02** ^**+**^	1.41E-09^-^	1.42E-09^-^	1.60E-09^-^	1.89E-02^-^	-
F16	Mean	5.00E+00	2.00E+00	1.00E+00	3.00E+00	3.00E+00	4.00E+00
	Std	1.97E-01	1.78E+00	6.56E-01	3.83E-01	3.08E-01	2.86E-01
	p-value	**1.41E-09** ^**+**^	3.07E-04^-^	1.04E-08^-^	4.54E-07^-^	5.50E-03^-^	-
F17	Mean	7.80E+02	1.98E+02	1.39E+03	8.40E+02	5.50E+02	4.87E+02
	Std	4.44E+01	1.17E+01	1.98E+02	1.07E+02	7.75E+01	6.35E+01
	p-value	**1.41E-09** ^**+**^	1.42E-09^-^	**1.42E-09** ^**+**^	**1.80E-09** ^**+**^	**2.80E-03** ^**+**^	-
F18	Mean	9.10E+02	3.09E+02	1.51E+03	2.53E+03	1.20E+03	8.90E+02
	Std	1.94E+01	2.00E+02	2.11E+02	2.25E+02	1.87E+02	1.59E+01
	p-value	**2.50E-03** ^**+**^	1.60E-09^-^	**1.42E-09** ^**+**^	**1.42E-09** ^**+**^	**1.99E-07** ^**+**^	-
F19	Mean	1.10E+01	1.80E+01	5.70E+02	8.20E+01	4.90E+01	1.10E+01
	Std	1.49E+00	4.63E+00	6.71E+02	1.27E+01	1.11E+01	1.29E+00
	p-value	5.35E-01 ^=^	**5.21E-09** ^**+**^	**1.41E-09** ^**+**^	**1.41E-09** ^**+**^	**1.41E-09** ^**+**^	-
F20	Mean	4.99E+01	5.00E+01	4.99E+01	4.99E+01	5.00E+01	4.95E+01
	Std	4.81E-01	0.00E+00	2.24E-01	0.00E+00	0.00E+00	2.66E-09
	p-value	**4.28E-02** ^**+**^	**1.04E-02** ^**+**^	3.97E-01 ^=^	1.18E-01 ^=^	**1.04E-02** ^**+**^	-
F21	Mean	3.70E+02	4.10E+02	4.40E+02	4.20E+02	3.30E+02	3.80E+02
	Std	4.49E+01	3.95E+01	4.69E+01	2.97E+01	5.48E+01	3.67E+01
	p-value	3.17E-01 ^=^	**5.12E-07** ^**+**^	**4.39E-09** ^**+**^	**5.17E-08** ^**+**^	5.86E-01 ^=^	-
F22	Mean	1.40E+03	5.48E+03	1.56E+04	5.05E+03	6.97E+03	2.15E+03
	Std	4.01E+02	1.12E+03	1.30E+03	6.52E+02	9.59E+02	5.34E+02
	p-value	8.86E-06^-^	**1.42E-09** ^**+**^	**1.42E-09** ^**+**^	**1.42E-09** ^**+**^	**1.42E-09** ^**+**^	-
F23	Mean	2.88E+04	1.10E+04	2.01E+04	2.57E+04	2.38E+04	2.15E+04
	Std	6.13E+02	1.37E+03	1.13E+03	4.04E+03	5.09E+03	1.05E+04
	p-value	1.04E-01 ^=^	2.98E-02^-^	2.98E-02^-^	6.55E-01 ^=^	7.86E-01 ^=^	-
F24	Mean	3.00E+02	3.70E+02	5.80E+02	6.10E+02	5.50E+02	2.10E+02
	Std	2.17E+01	1.83E+01	2.15E+01	2.30E+01	2.26E+01	2.49E+01
	p-value	**2.57E-09** ^**+**^	**1.80E-09** ^**+**^	**1.42E-09** ^**+**^	**1.42E-09** ^**+**^	**1.42E-09** ^**+**^	-
F25	Mean	4.10E+02	4.60E+02	7.10E+02	6.00E+02	6.30E+02	3.70E+02
	Std	1.41E+01	1.58E+01	2.09E+01	1.95E+01	1.88E+01	1.47E+01
	p-value	**2.87E-08** ^**+**^	**1.42E-09** ^**+**^	**1.42E-09** ^**+**^	**1.42E-09** ^**+**^	**1.42E-09** ^**+**^	-
F26	Mean	3.90E+02	4.40E+02	5.40E+02	6.80E+02	6.20E+02	3.10E+02
	Std	1.81E+01	1.27E+01	1.33E+02	1.36E+01	2.24E+01	1.35E+01
	p-value	**1.60E-09** ^**+**^	**1.42E-09** ^**+**^	**5.91E-05** ^**+**^	**1.42E-09** ^**+**^	**1.42E-09** ^**+**^	-
F27	Mean	1.33E+03	1.99E+03	3.75E+03	4.45E+03	3.56E+03	8.80E+02
	Std	1.64E+02	1.12E+02	1.44E+02	1.99E+02	1.65E+02	1.69E+02
	p-value	**6.80E-07** ^**+**^	**2.87E-08** ^**+**^	**1.42E-09** ^**+**^	**1.42E-09** ^**+**^	**1.42E-09** ^**+**^	-
F28	Mean	2.89E+03	3.38E+03	8.90E+03	1.76E+04	4.72E+03	2.77E+03
	Std	7.82E+02	9.91E+02	1.25E+03	1.28E+03	1.36E+03	1.07E+03
	p-value	**8.87E-05** ^**+**^	**4.67E-06** ^**+**^	**1.41E-09** ^**+**^	**1.41E-09** ^**+**^	**1.99E-07** ^**+**^	-
w/l/t		18/5/5	20/7/1	23/3/2	24/2/2	22/3/3	-

### Sensors arrayed by proposed LCSO

To optimize the umpiring system, the number of sensor nodes should be minimized by LCSO under the premise that the detection range of the designed intelligent umpiring system covers the entire tabletop. The maximum detection distance (*R*_*m*_) of the sensor is a key factor for the final result of sensor array optimization, which is decided by the resolution of the sensor and the minimal vibration caused by the table tennis hitting the table during the competition. Obviously, the table will generate a minimal vibration when the ball hits the top of the net and then drops in free fall. The instantaneous velocity (*v*_1_) of the ball when colliding with the table can be defined as:

$$v_{\text{1}}=\sqrt{2gh}$$ (11)

Where *h* is the height of net, *g* is the acceleration of gravity. We set downward direction as the positive reference direction, the vibration intensity (*a*_1_) at the collision point is given by:

$$a_{1}=\frac{\left(m_{\text{1}}v_{\text{1}}+m_{1}v_{\text{2}}\right)S_{2}}{t_{c}S_{1}m_{2}}$$ (12)

Where *m*_1_ is the weight of the table tennis ball, *t*_*c*_ and *S*_1_ are the contact time and the maximum contact area between the ball and the table respectively, *m*_2_ and *S*_2_ are the weight and area of the table respectively. *v*_2_ is the rebound velocity of the ball, defined as following:

$$v_{2}=COR\times v_{1}$$ (13)

Where COR is the coefficient of restitution [51].

To get the maximum detection distance (*R*_*m*_), we have carried out the following equation derivation process.

The transfer process of mechanical vibration inside the ping pong table is analyzed by elastic dynamics method. The physical equation, geometric equation and balance equation in polar coordinates are established as Eqs (20–22).


$$physical\;equation:\left\{\begin{array}{c} \varepsilon_{r}=\frac{1}{E}\left[\sigma_{r}-\mu (\sigma_{\theta }+\sigma_{z})\right]\\ \varepsilon_{\theta }=\frac{1}{E}\left[\sigma_{\theta }-\mu (\sigma_{r}+\sigma_{z})\right]\\ \varepsilon_{z}=\frac{1}{E}\left[\sigma_{z}-\mu (\sigma_{r}+\sigma_{\theta })\right]\\ \gamma_{zr}=\frac{2(1+\mu )}{E}\tau_{zr} \end{array}\right.$$ (14)

$$geometric\;equation:\left\{\begin{array}{c} \varepsilon_{r}=\frac{\partial u}{\partial r}\\ \varepsilon_{\theta }=\frac{u}{r}\\ \varepsilon_{z}=\frac{\partial w}{\partial z}\\ \gamma_{zr}=\frac{\partial u}{\partial z}+\frac{\partial w}{\partial r} \end{array}\right.$$ (15)

$$balance\;equation:\rho \frac{\partial^{2}u}{\partial t^{2}}=\frac{\partial \sigma_{r}}{\partial r}+\frac{\partial \tau_{zr}}{\partial z}+\frac{\sigma_{r}-\sigma_{\theta }}{r}+R$$ (16)

Where *u*, *w* are the displacement components of one point in the ping pong table in the r-axis and z-axis directions. *σ*_*θ*_, *σ*_*r*_ and *σ*_*z*_ are the normal stresses in the *θ*-axis, r-axis and z-axis directions respectively. *τ*_*zr*_ is a upward shear stress perpendicular to the zr-plane. *ε*_*θ*_, *ε*_*r*_ and *ε*_*z*_ are the normal strains in the *θ*-axis, r-axis and z-axis directions respectively. *γ*_*zr*_ is a upward shear strain perpendicular to the zr-plane. R is the periodic stress along the r-axis applied to the outer layer of the table. M and K are frequency and amplitude of the vibration source. The constants E, ρ, μ represent the Young’s modulus, density and Poisson’s ratio of the table material, respectively.

To simplify the model, make *R* = 1*e*8 × sin(20*πt*), z = 0. After substituting the geometric equations into the physical equations, we can obtain:

$$\left\{\begin{array}{c} (1+\mu )\sigma_{r}=\frac{\mu E}{1-2\mu }(\frac{\partial u}{\partial r}+\frac{u}{r}+\frac{\partial w}{\partial z})+E\frac{\partial u}{\partial r}\\ (1+\mu )\sigma_{\theta }=\frac{\mu E}{1-2\mu }(\frac{\partial u}{\partial r}+\frac{u}{r}+\frac{\partial w}{\partial z})+E\frac{u}{r}\\ (1+\mu )\sigma_{z}=\frac{\mu E}{1-2\mu }(\frac{\partial u}{\partial r}+\frac{u}{r}+\frac{\partial w}{\partial z})+E\frac{\partial w}{\partial z}\\ \gamma_{zr}=\frac{\partial u}{\partial z}+\frac{\partial w}{\partial r} \end{array}\right.$$ (17)

As z approaches infinity, we can ignore the axial displacement *w*, shear stress *τ*_*zr*_, and the axial time-domain equilibrium equation. Then the equation set-up (17) can be reduced to the equation set-up (18).


$$\left\{\begin{array}{c} (1+\mu )\sigma_{r}=\frac{\mu E}{1-2\mu }(\frac{\partial u}{\partial r}+\frac{u}{r})+E\frac{\partial u}{\partial r}\\ (1+\mu )\sigma_{\theta }=\frac{\mu E}{1-2\mu }(\frac{\partial u}{\partial r}+\frac{u}{r})+E\frac{u}{r}\\ \left(1+\mu )\sigma_{z}=\frac{\mu E}{1-2\mu }(\frac{\partial u}{\partial r}+\frac{u}{r}\right)\\ \gamma_{zr}=\frac{E}{2(1+\mu )}(\frac{\partial u}{\partial z}+\frac{\partial w}{\partial r})=0 \end{array}\right.$$ (18)

Substitute equation set-up (18) into equation set-up (15) to get:

$$\left\{\begin{array}{c} \rho \frac{\partial^{2}u}{\partial t^{2}}-K\sin(Mt)=A(\frac{\partial^{2}u}{\partial r^{2}}+\frac{1}{r}\frac{\partial u}{\partial r}-\frac{u}{r^{2}})\\ \left(A=\frac{(1-u)E}{\left(1-2u)(1+u\right)},K=10^{8}\right) \end{array}\right.$$ (19)

According to the separation of variables method, assume *u* = *y*(*r*) × sin(*Mt*), then Eq (19) can be simplified to Eq (20), and its analytical Eq (21) can be obtained through MATLAB.


$$Ay^{''}+Ay^{'}-A\frac{y}{r^{2}}+\rho M^{2}y+K=0$$ (20)

$$y(r)=-\frac{K\pi }{2\rho M^{2}}struveH(1,Mr\sqrt{\frac{\rho (1-2\mu )(1+\mu )}{E(1-\mu )}})$$ (21)

Where *struveH*(1, *x*) is an intrinsic function of software Mathematica. And *struveH*(*n*, *x*) has branch-cut discontinuity in the range of −∞~0 on the complex plane. For some specific variable values, *struveH* can automatically calculate the exact value and obtain the value of any numerical precision.

Finally, *R*_*m*_ will be obtained by solving the equation set-up (22) [52].


$$\left\{\begin{array}{l} y(r)=-\frac{K\pi }{2\rho M^{2}}struveH(1,Mr\sqrt{\frac{\rho (1-2\mu )(1+\mu )}{E(1-\mu )}})\\ \frac{1}{2}a_{1}{\left(\frac{t_{c}}{2}\right)}^{2}=x\left(0\right)\\ \frac{1}{2}a_{2}{\left(\frac{t_{c}}{2}\right)}^{2}=x\left(R_{m}\right) \end{array}\right.$$ (22)

Where *StruveH* is a function of software Mathematica. *E*, *ρ*, and *μ* represent the table’s Young’s modulus, density, and Poisson’s ratio, respectively. *M* and *K* denote the frequency and the amplitude of the external stress, respectively. *r* is the propagation distance of the vibration. *a*_2_ is the resolution of the sensor.

**Fig 5:**
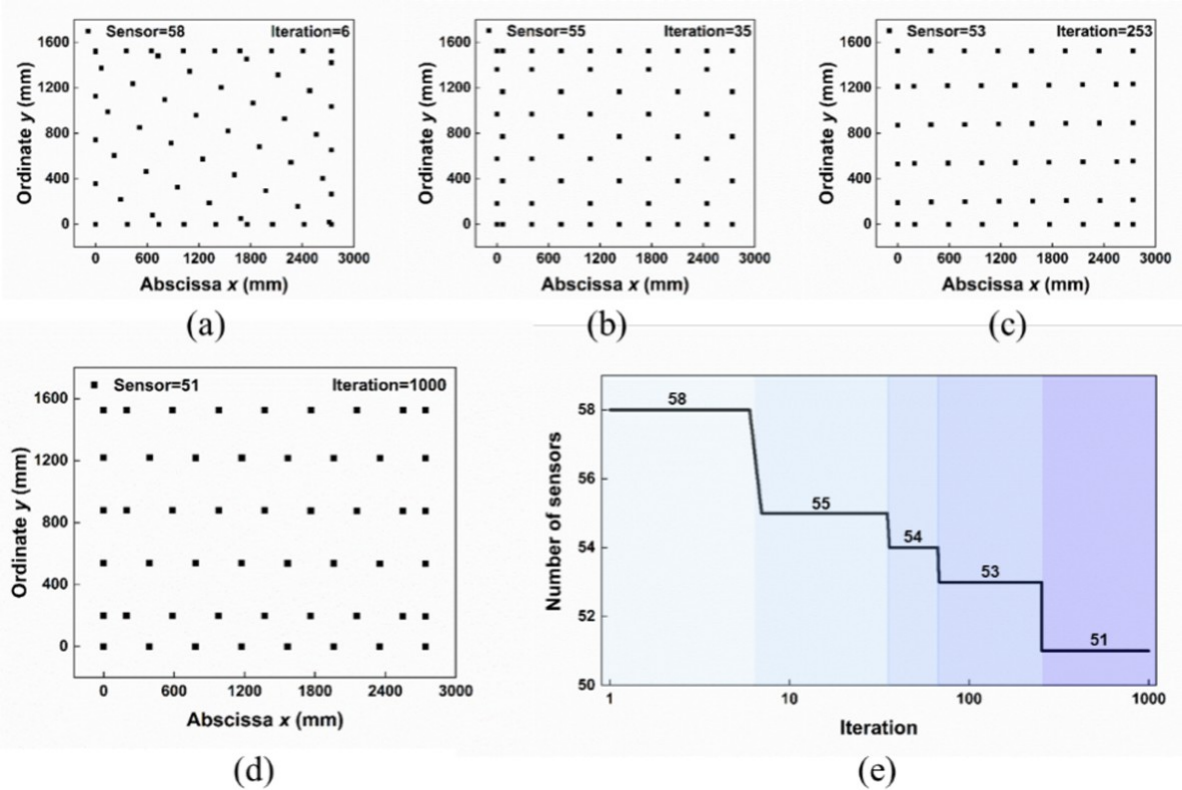
Best sensor distribution in different iteration ((a) 6, (b) 35 and (c) 253) swarms. (d) The optimal sensor distribution after LCSO optimization. (e) The number of sensors with different iterations.

Based on the designed model, the sensors distribution optimization has been converted to search the minimum value of the function *minsensor* = *f* (*α*, *β*, *x*, *y*) via LCSO. In this work, each particle in the LCSO represents a candidate solution and the return value of the function *minsensor* = *f* (*α*, *β*, *x*, *y*) represents particle’s fitness. The position of the particle is expressed in discrete form and divided into 4 parts, representing *α*, *β*, *x*, and *y*. During the swarm iteration, the particles that lose the competition learn from the winner and the higher-level particles and update their position and velocity. The winner and the highest-level particles directly enter the next generation. The parameters involved in the experiment are listed in Table 3. Fig 5A–5C show the sensor distribution in different iteration swarms. After LCSO optimization and 1000 iterations, the number of the sensors reduces to 51 (Fig 5D). Overall, the relationship between number of sensors and time of iteration is shown in Fig 5E. It can be observed that the number of sensors decreases gradually as the number of iterations increases. These test results illustrate that the minimum number of sensors is successfully obtained by LCSO, and the optimal solution (*α* = 60.012°, *β* = 90.0880°, *x* = 1371, *y* = 197) meets the expectation of the largest triangle area.

**Table 3:** Parameters applied in sensors distribution by LCSO.

Symbol	Quantity	Value
*NP*	Swarm size	100
*D*	Particle dimension	40
*R*_*m*_	Maximum detection distance	39.25 cm
*c*_*1*_	Acceleration coefficients	1
*c*_*2*_	Acceleration coefficients	0.5
*h*	Net hight	15.25 cm
*m*_*1*_	Ball weight	2.53 g
*m*_*2*_	Table weight	118 kg
*S*_*1*_	Maximum contact area	50.89 mm^2^
*S*_*2*_	Table surface area	4.18 m^2^
*t*_*c*_	Contact time	0.4192 ms
*a*_*2*_	Sensor resolution	0.1 m/s^2^
*E*	Young’s modulus	2.2 GPa
*K*	External stress amplitude	20.3 N
*ρ*	Density	880 Kg/m^3^
*μ*	Poisson’s ratio	0.24

**Fig 6:**
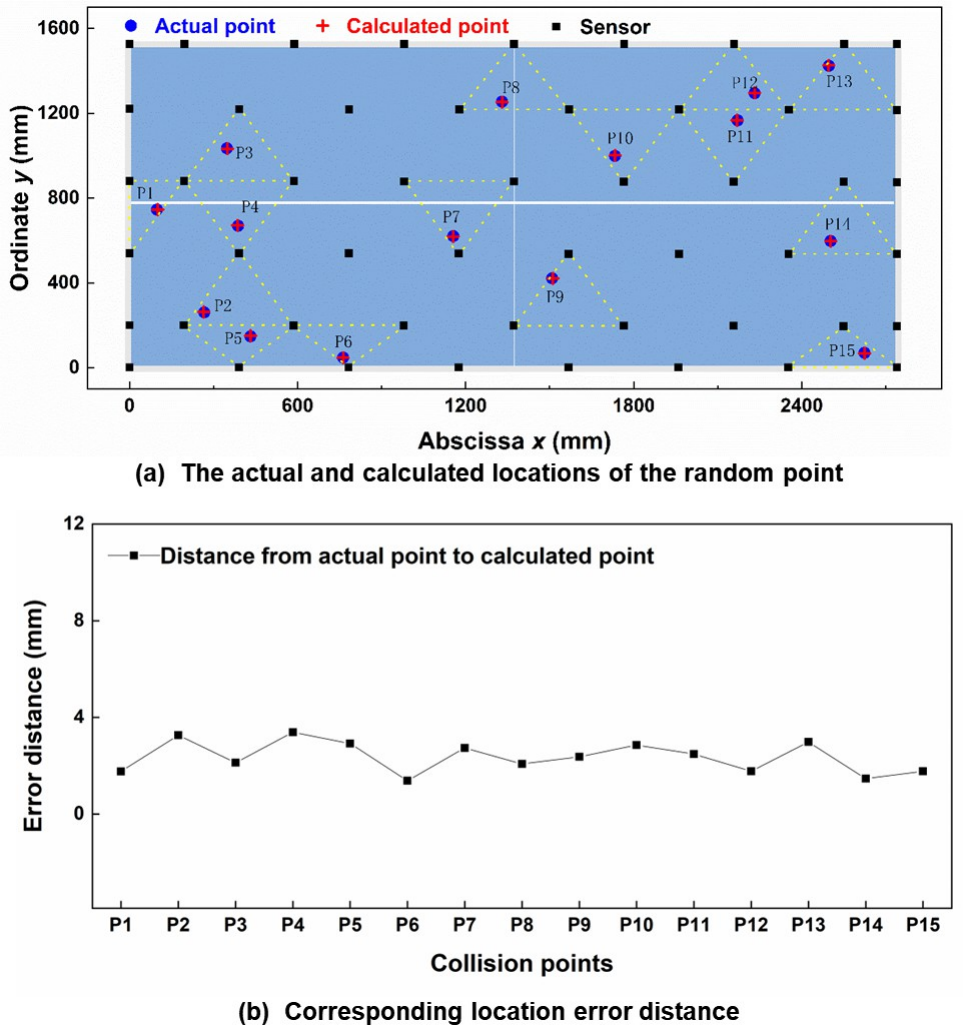
(a) Comparison of the calculated location and actual location of the random point and (b) corresponding location error.


The reliability of the designed intelligent umpiring system is crucial for its application. To verify this performance, we randomly generate some points on the table tennis table by Matlab and calculate the coordinates of all points by the designed umpiring system. Based on the final sensor distribution (Fig 5D), the random points and their three nearest sensors are shown in Fig 6A. The coordinate (*x*_*n*_, *y*_n_) of each point can be obtained by solving equation set-up (23).

$$\left\{\begin{array}{c} {\left(x_{an}-x_{n}\right)}^{2}+{\left(y_{an}-y_{n}\right)}^{2}=r_{an}{}^{2}\\ {\left(x_{bn}-x_{n}\right)}^{2}+{\left(y_{bn}-y_{n}\right)}^{2}=r_{bn}{}^{2}\\ {\left(x_{cn}-x_{n}\right)}^{2}+{\left(y_{cn}-y_{n}\right)}^{2}=r_{cn}{}^{2} \end{array}\begin{array}{cc} & \left(n=1,2,\cdot \cdot \cdot ,15\right) \end{array}\right.$$ (23)

**Fig 7:**
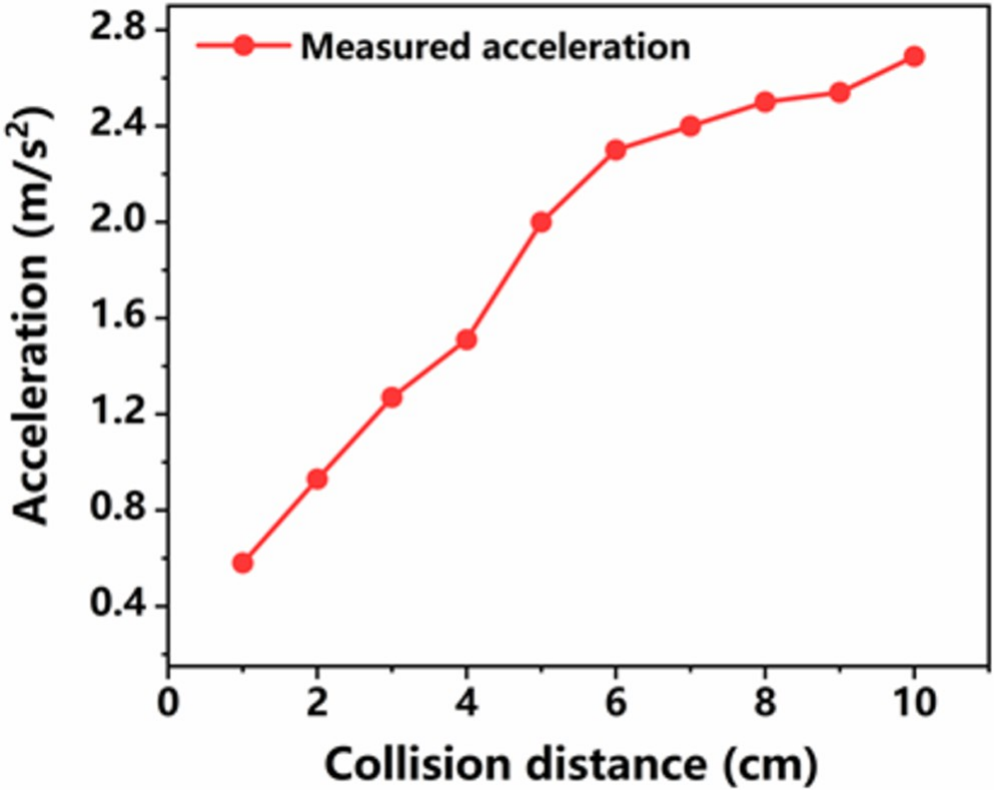
The measured acceleration by freeing the Ping-Pong ball with different collision distance.

Where *n* is the serial number of random point, (*x*_*an*_, *y*_*an*_), (*x*_*bn*_, *y*_*bn*_), and (*x*_*cn*_, *y*_*cn*_) are the coordinates of the three nearest sensors respectively, *r*_*an*_, *r*_*bn*_, and *r*_*cn*_ are distances between the point *P*_*n*_ to each of its three nearest sensors. The parameters involved in equation set-up (23) are listed in Table 4. The corresponding experimental results are shown in Fig 6A and 6B. All points on the table are accurately located and the location errors are less than 3.39 mm. This proves that our umpiring system has accurate positioning and high stability. In addition, to deeply understand the actual collision output performance of the sensor, we also measured the output voltage of the self-powered acceleration sensor when the sensor sensed actual collision point made by table tennis. In our previous study, we have tested the sensitivity of the sensor and the sensitivity of the sensor is up to 20.4 V/(m/s^2^) when the range of acceleration is 1–11 m/s^2^ [25]. The relationship between acceleration and collision distance is shown in Fig 7. The measured acceleration increases with the increasing collision distance. This proves that our sensor has a superior linear output.

**Table 4:** Parameters applied in equation set-up (23).

Point 1	ra1	229	Nearest three sensors	Sensor A1 (0,539)
	rb1	165		Sensor B1 (0,879)
	rc1	163		Sensor C1 (195,978)
Point 2	ra2	95	Nearest three sensors	Sensor A2 (194,199)
	rb2	288		Sensor B2 (390,0)
	rc2	325		Sensor C2 (586,198)
Point 3	ra3	870	Nearest three sensors	Sensor A3 (586,198)
	rb3	850		Sensor B3 (194,199)
	rc3	498		Sensor C3 (391,538)
Point 4	ra4	131	Nearest three sensors	Sensor A4 (391,538)
	rb4	284		Sensor B4 (195,878)
	rc4	288		Sensor C4 (587,878)
Point 5	ra5	767	Nearest three sensors	Sensor A5 (195,878)
	rb5	746		Sensor B5 (587,878)
	rc5	1070		Sensor C5 (392,1218)
Point 6	ra6	51	Nearest three sensors	Sensor A6 (782,0)
	rb6	232		Sensor B6 (586,198)
	rc6	262		Sensor C6 (979,197)
Point 7	ra7	48	Nearest three sensors	Sensor A7 (1176,537)
	rb7	344		Sensor B7 (980,877)
	rc7	366		Sensor C7 (1372,877)
Point 8	ra8	157	Nearest three sensors	Sensor A8 (1177,1217)
	rb8	242		Sensor B8 (1569,1216)
	rc8	512		Sensor C8 (1766,1525)
Point 9	ra9	258	Nearest three sensors	Sensor A9 (1371,197)
	rb9	348		Sensor B9 (1764,196)
	rc9	133		Sensor C9 (1568,536)
Point 10	ra10	127	Nearest three sensors	Sensor A10 (1765,876)
	rb10	272		Sensor B10 (1569,1216)
	rc10	315		Sensor C10 (1962,1216)
Point 11	ra11	292	Nearest three sensors	Sensor A11 (2157,875)
	rb11	214		Sensor B11 (1962,1216)
	rc11	189		Sensor C11 (2354,1215)
Point 12	ra12	393	Nearest three sensors	Sensor A12 (2551,1525)
	rb12	281		Sensor B12 (1962,1216)
	rc12	145		Sensor C12 (2354,1215)
Point 13	ra13	245	Nearest three sensors	Sensor A13 (2354,1215)
	rb13	332		Sensor B13 (2740,1214)
	rc13	277		Sensor C13 (2740,1525)
Point 14	ra14	162	Nearest three sensors	Sensor A14 (2353,535)
	rb14	245		Sensor B14 (2740,535)
	rc14	280		Sensor C14 (2550,875)
Point 15	ra15	135	Nearest three sensors	Sensor A15 (2740,0)
	rb15	280		Sensor B15 (2352,0)
	rc15	145		Sensor C15 (2549,195)

## Conclusion

In summary, we presented a real-time intelligent umpiring system based on arrayed self-powered acceleration sensor nodes to assist referee to make precise umpiring. To avoid the periodic charging and reduce the number of sensors, high sensitivity self-powered acceleration sensors were installed on the back surface of the table tennis table after arrayed sensor nodes conducted a novel particle swarm optimization (LCSO). A model was established to optimize sensor nodes distribution. To minimize the function *minsensor* = *f* (*α*, *β*, *x*, *y*) based on the model, we presented an improved particle swarm optimization—LCSO. The number of sensors reduced from 58 to 51 via the LCSO algorithm. The simulation results on Matlab showed that the designed intelligent umpiring system had a high accuracy (errors below 3.5 mm). This work proposed a novel sensor positioning model and an effective method to optimize sensor nodes, which would make the umpiring of the table tennis competition precise and real-time.

Although the proposed algorithm showed competitive performance on both numerical comparison and application, the experimental results showed that the proposed algorithm still left room for further improvement. This can be explained by “No free lunch” theory. Thus, in our future work, we will continue to study the proposed algorithm with respect to specific problems. Targeting different kinds of problem, we will design adaptive parameters to improve the robustness of LCSO.
